# Impact of intermittent fasting versus vitamin D on high fat fructose-induced pancreatic steatosis: possible role of aquaporins

**DOI:** 10.1186/s10020-025-01239-w

**Published:** 2025-05-26

**Authors:** Basma Adel Khattab, Maha Osman Hammad, Zienab Helmy Eldken, Doaa Hellal, Sherin Zohdy Mohamed, Noha Hammad Sakr

**Affiliations:** 1 Department of Anatomy and Embryology, Faculty of Medicine, Mansoura University, Mansoura, Egypt; 2 Department of Medical Biochemistry and Molecular Biology, Faculty of Medicine, Mansoura University, Al-Daqhalia Governorate, P.O.No.35516, Mansoura, Egypt; 3 Department of Medical Physiology, Faculty of Medicine, Mansoura University, Mansoura, Egypt; 4 Department of Clinical Pharmacology, Faculty of Medicine, Mansoura University, Mansoura, Egypt; 5 Department of pharmacology, College of medicine, University of Ha’il, Ha’il, Saudi Arabia; 6 Department of Internal medicine, Faculty of Medicine, Horus University, New Damietta, Egypt; 7 Department of Anatomy and Embryology, Faculty of Medicine, KafrElsheikh University, KafrElsheikh, Egypt

**Keywords:** Pancreatic steatosis, Aquaporin, NLRP3, Fasting, Vitamin D

## Abstract

**Background:**

The molecular basis of pancreatic steatosis is not entirely known. Aquaporins (AQPs) are integral membrane proteins involved in a variety of pancreatic functions. Given the little data regarding the potential role of aquaporins in the pathogenesis of pancreatic steatosis, this study was designed to assess the role of aquaporins and the NLRP3-inflammasome in the rat model of high-fat fructose diet (HFFD) and to investigate the impact of vitamin D supplementation and alternate day fasting (ADF) in ameliorating HFFD-induced pancreatic steatosis.

**Method:**

Twenty-four Sprague-Dawley male rats were divided equally into 4 groups. Group I (control group), Group II (HFFD group), Group III (HFFD + ADF group), and Group IV (HFFD + vitamin D group). By the end of the experiment, fasting blood samples were collected for determination of blood glucose, serum insulin, lipid profile, and insulin resistance. Oxidative stress biomarkers (malondialdehyde and reduced glutathione), inflammatory markers (interleukin-1β and TNF-α), and expression of aquaporins (AQP-1, AQP-3, and AQP-7) genes were evaluated in pancreatic tissues. Histopathological examination of the pancreas and immunohistochemistry of the NLRP3-infammasome and AQP-7 were performed.

**Results:**

The HFFD group exhibited pancreatic steatosis with a significant elevation in the levels of blood sugar, serum insulin, insulin resistance, lipid profile, oxidative stress, inflammatory markers, and AQP-3 and AQP-7 mRNA expressions. Regarding histopathology, there were pale vacuolated-stained cytoplasm in acinar pancreatic cells and increased immunoreactivity for AQP-7 and NLRP3-inflammasome. All these parameters improved with ADF and vitamin D supplementation, with more favorable effects for ADF.

**Conclusion:**

ADF and vitamin D treatment ameliorated the effect of the high-fat fructose diet at both levels of the biochemical and histopathological examinations.

## Background

Fatty pancreas is ectopic fat deposition in the pancreas and is linked to elevated visceral fat levels, free fatty acids, triglycerides, cholesterol, low-density lipoprotein, and insulin resistance (IR) (Neeland et al. 2019). Two processes lead to the deposition of fat inside the pancreas: either fatty deposition following acinar cell death or fatty infiltration, which is related to obesity and metabolic syndrome (Sreedhar et al. 2020). The fructose element of sugar may be very detrimental, and high fructose diets rapidly trigger all the critical metabolic syndrome symptoms (Marinho et al. 2019).

Fatty infiltration and subsequent inflammation lead to notable changes in pancreatic morphology, including adipocyte accumulation within the parenchyma, fibrosis, immune cell infiltration, and atrophy of acinar cells, all of which contribute to disrupted pancreatic architecture and impaired function (Lilly et al. 2023).

HFFD is known to induce significant metabolic disturbances, leading to increased oxidative stress and inflammation. Excessive intake of fat and fructose promotes the overproduction of reactive oxygen species (ROS), overwhelming the antioxidant defense system and resulting in lipid peroxidation and oxidative damage to tissues. This oxidative stress plays a critical role in the pathogenesis of insulin resistance, hepatic steatosis, and other metabolic syndromes. Simultaneously, HFFD stimulates pro-inflammatory signaling pathways, such as NF-κB and JNK, leading to elevated levels of inflammatory cytokines like TNF-α, IL-6, and IL-1β, which further aggravate tissue damage and metabolic dysfunction (Ekta et al. 2020).

Aquaporins (AQPs) are transmembrane water channel proteins that help move water across cell membranes quickly. Aquaglyceroporins (AQP7, AQP9, AQP10, AQP11, and AQP3) are permeable to glycerol, urea, and water, while the unorthodox aquaporins (AQP12 and AQP11) still have unproven permeability (Arsenijevic et al. 2019).

AQPs are expressed in both the exocrine and endocrine pancreas, and they have a crucial role in vital physiological processes associated with the secretion of insulin (da Silva et al. 2018). Pancreatic ductal cells and acini, which are related to the secretion of fluid, have high expression of the water channel AQP1 (Venglovecz et al. 2018).

AQP7, an aquaglyceroporin that is abundantly expressed in pancreatic ß-cells (Calamita et al. 2018). In AQP7 deletion mice, ß-cells showed reduced size and mass, increased triglyceride and glycerol contents, decreased insulin content, and increased glycerol kinase activity with decreased release of glycerol on stimulation of lipolysis, according to a prior study (Calamita and Delporte 2021).

Although direct studies on HFFD-induced effects on pancreatic aquaporins are limited, recent findings suggest that such regimens induce oxidative stress, insulin resistance, and systemic inflammation, which may contribute to the downregulation of AQP1 in the testis (Marchiani et al. 2015). Moreover, Elhessy et al. (2024) demonstrated that the hypertrophic effect seen in rat jejunum villi and crypts stimulated by HFFD and the metabolic stress modulates aquaporin expression leading to a significant increase in AQP1, AQP3, and AQP7 expression.

NLRP3-inflammasome is one of the multimeric protein complexes of the leucine-rich repeat (NLR)-containing pyrin, nucleotide-binding domain, and HIN domain family. NLRP3 can activate caspase-1, which is responsible for cleavage of inactive pro-interleukin (IL)-1β and pro-IL-18 into their active forms. A well-known pro-inflammatory cytokine, IL-1β, can trigger the secretion of additional pro-inflammatory mediators, such as IL-6 and TNF-α, starting a cascade of pro-inflammatory markers that participate in the occurrence of metabolic syndrome (Arend et al. 2008). Systemic, low-grade persistent inflammation is a characteristic of insulin resistance and is one of the effects of NLRP3-inflammasome activation (Wani et al. 2021).

NLRP3 stimulation is linked to a variety of water-permeable AQPs. Following NLRP3 stimulation by hypotonicity or ATP therapy, AQP1 deficiency decreases the production of IL-1β (Compan et al. 2012). The AQP3 has a vital role in the inflammatory reaction by releasing IL-1β and stimulating the NLRP3-inflammasome (da Silva et al. 2021). HFHF diet induces metabolic disorders, oxidation, and inflammation, as well as changes in islet size and irregular secretory functions in the pancreas. HFHF diet-induced inflammation occurred after the accumulation of oxidative stress, suggesting an avenue to prevent improper diet-related diabetes (Zhao et al. 2022).

Intermittent fasting (IF) increases the activity of defense mechanisms in cells, including autophagy, mitochondrial function, and stress response pathways. Additionally, it was shown to regulate the circadian rhythms of hormones like leptin and insulin(Joaquim et al. 2022). Through various mechanisms, 1, 25-dihydroxy vitamin D is vital for maintaining glucose homeostasis. It boosts and improves β-cell function in addition to improving insulin sensitivity in the target cells (skeletal muscle, adipose tissue, and liver) (Jin et al. 2018). Because the treatments for the side effects of the HFFD are limited, the potential of ADF and vitamin D supplementation as treatments for the metabolic consequences of a HFFD still needs more extended studies to fully understand their effects and optimize their use.

The purpose of this study was to examine the role of the NLRP3 inflammasome and aquaporins in pancreatic function in normal and high-fat fructose-fed rats. It also investigated the effect of alternate-day fasting and vitamin D supplementation on ameliorating high-fat fructose diet-induced pancreatic changes, particularly in histopathology, glucose homeostasis, lipid profile, and inflammatory and oxidative stress biomarker levels.

## Material & methods

### Sample size

The sample size was calculated using the Power Analysis Sample Size software program (PASS) version 15.0.5 for Windows. A previous study performed by Elhessy et al. (2024) reported that the mean (SD) of the difference of level of relative quantification of gene expression of AQP-7 between the control group and the high fat diet group was 2.04 (0.062). A minimal sample size of 6 rats in each group is required in each group, with an expected difference of 0.1, a power of 80%, at the 5% significance level. The following equation was used: n = (Z_α_/2 + Z_β_)2 *2*σ2 / d2.

**n** = sample size.

**Z**_**α**_**/2** = 1.96 (the critical value that divides the central 95% of the Z distribution from the 5% in the tail).

**Z**_**β=**_ is the critical value of the normal distribution at β (for a power of 80%, β is 0.2 and the critical value is 0.84).

**σ**^**2**^ **=** is the population variance.

**d** = is the mean difference **= 0.1**.

### Experimental design and animals

Twenty-four Sprague-Dawley male rats weighing 150–180 g were purchased from the Animal House at Faculty of Medicine, Kafrelsheikh University and housed in cages with a 12:12-h light/dark cycle at room temperature of 18–22◦C and under controlled conditions for humidity. Ethical approval of this study was obtained from the Faculty of Medicine, Kafrelsheikh University, Egypt (approval code: MKSU 50-7-3). Animals (at the age of 6 weeks) were randomly assigned into four groups:

- Group I: (Control group) (*n* = 6): animals fed on water ad libitum and basal meal (55% carbohydrates, including 3% sucrose + 10% fat, 3-3.5 kcal/g) for 12 weeks.
- Group II: (HFFD group) (*n* = 6): animals fed on high-fat meal (5% in basal diet + 40% animal fat) (45% fat) (Jensen et al. 2018), 20% fructose, and water ad libitum for 12 weeks (Mamikutty et al. 2014).
- Group III (HFFD and ADF group) (*n* = 6): rats fed on HFFD (45% fat, 20% fructose) for 12 weeks, followed by the alternative day fasting (ADF) protocol (24-hour feeding, then 24-hour fasting) from the beginning of the 9th week until the end of the study (Xu et al. 2022).
- Group IV: (HFFD and vitamin D group) (*n* = 6): rats fed on HFFD (45% fat, 20% fructose) for 12 weeks. Animals were given intra-peritoneal injections of 5 µg⁄kg of 1, 25 (OH)_2_ D_3_ each 2 days with their prior diets from the beginning of the 9th week till the end of the study (Yin et al. 2012).

### Blood samples and tissue specimens’ collection

A cervical dislocation was used for the rat’s scarification at the end of the study. 3% isoflurane narcosis for 1 min before cervical dislocation to ensure the animal is fully unconscious. Cardiac puncture was done to collect fasting blood samples (for 12 h) in tubes without adding EDTA. Samples were allowed to clot for 10 min. For serum separation, centrifugation was performed, and samples were stored at − 20ºC until being analysed.

The pancreas was divided into three portions: the first was homogenized in 0.05 M ice-cold potassium phosphate buffer (pH; 7.5) using a tissue homogenizer at 40 °C, then centrifuged at 4,000 rpm for 15 min, and the supernatant was collected and stored on ice for the determination of inflammatory and oxidative stress biomarkers; the second was collected in RNAlater (100 µl RNAlater per 10 mg tissue) (Qiagen, Germany), placed at 4 °C for 1 day, and then stored at − 80 °C for analysis of gene expression; and the last one was fixed in 10% neutral buffered formalin to perform the histopathological and immunohistochemical analysis.

### Determination of serum lipid profile and fasting blood glucose (FBG)

FBG, total cholesterol (TC), LDL-cholesterol (LDL-C), triglycerides (TGs), and HDL-cholesterol (HDL-C) were estimated using commercial colorimetric endpoint kits (Biodiagnostics, Egypt) following the instructions of the manufacturer’s by using the Erba CHEM-7 device (ERBA Diagnostics, India).

### Measurement of serum insulin and insulin resistance (IR)

Rat insulin ELISA kit (Cloud-Clone Corp., China) was used to measure insulin concentrations following the instructions of the manufacturer’s depending on the technique of competitive inhibition enzyme immunoassay, absorbance was measured at 450 nm. Using a ChroMate 4300 microplate reader (Awareness Technologies, USA).

Insulin resistance (IR) was estimated via the Homeostasis Model Assessment Index (HOMA-IR): HOMA-IR = Insulin (µU/ml) X glucose (mg/dl) / 405 (Okita et al. 2013).

### Measurement of IL-1β and TNF-α in pancreatic tissues

Levels of IL-1β and TNF-α in pancreatic tissues were assessed using rat TNF-α and rat IL-1β ELISA kits (LifeSpan Biosciences, USA) following the instructions of the manufacturer’s based on the sandwich immunoassay principle. By utilizing a ChroMate 4300 microplate reader (Awareness Technologies, USA), the absorbance was measured spectrophotometrically.

### Measurement of markers of oxidative stress in pancreatic tissues

Reduced glutathione (GSH) and malondialdehyde (MDA) levels were estimated in pancreatic tissue homogenates to evaluate oxidative stress status using colorimetrically available kits (Biodiagnostic, Egypt) and according to the manufacturer’s guidelines. GSH levels were estimated depending on the reduction of 5,5’-dithiobis (dithionitrobenzoic acid) (DTNB) with GSH.

### Real time PCR for measuring mRNA expressions of AQP-1, AQP-3, and AQP-7

The modified protocol for the isolation of high-quality RNA from rat pancreas rich in RNase was used to isolate total tissue RNA (Hammad 2023) utilizing the QIAzol-reagent (Qiagen, Germany), and the next step was utilizing the Nanodrop (Thermo Fisher Scientific, USA) to assess the quality and quantity of RNA yield by estimating the optical density (OD) 260/280 ratios. Of total RNA, 1 µg from each sample was used to synthesize complementary DNA (cDNA) by the oligo dT-primed reverse transcriptase (RT) reaction using the SensiFAST™ cDNA Synthesis Kit according to the manufacturer’s instructions (Bioline, UK).

The cDNA fragments were then amplified by a real-time PCR apparatus (Applied Biosystems 7500, USA). PCR reactions consisted of 2 µl of cDNA products, 10 µl of HERA SYBR green PCR Master Mix (Willowfort, UK), 2 µl (10 pmol/µl) primer, and 6 µl of nuclease-free water in a total reaction volume of 20 µl/well. The PCR program performed for amplification of AQP-1, AQP-3, AQP-7, and glyceraldehyde-3-phosphate dehydrogenase (GAPDH) was initial denaturation at 98ºC for 2 min, followed by 10 s of 45 cycles at 95ºC and 30 s of another 45 cycles at 60ºC. Utilizing Primer3 software (v.4.1.0; http://primer3.ut.ee) primer sets were designed. (https://www.ncbi.nlm.nih.gov/tools/primer-blast/primertool) was used for checking the primer specificity. GAPDH was used as the endogenous reference gene. The frequency of the utilized primer pairs is supplied in Table 1. For the determination of non-specific products and primer dimers, all final real-time PCR product melting curves were analysed. The plots of amplification were analysed to get the cycle threshold (CT), which was utilized to measure the Relative Quantification (RQ) of the target mRNA expression levels, using the comparative threshold method (2^−ΔΔCT^) (Livak and Schmittgen 2001).

**Table 1:** Real-time PCR primer sequences for AQP-1,** AQP-3**,** AQP-7**,** and GAPDH**

Gene	Forward primers	Reverse primers	Product length	Reference sequence
**AQP-1**	CCCTCTTCGTCTTCATCAGC	CTGAGCCACCTAAGTCTCGG	438 bp	NM_012778.2
**AQP-3**	TGGACCTCGCCTTTTCACTG	GGAGCGTTTTTAGCCCGAGA	338 bp	NM_031703.2
**AQP-7**	GTAATGGAGGACCAGAAACAAG	TATGAGCCACGGAACCAAG	248 bp	NM_019157.2
**GAPDH**	CCTCGTCTCATAGACAAGATGGT	GGGTAGAGTCATACTGGAACATG	169 bp	NM_017008.4

### Histological and immunohistochemical staining of AQP-7 and NLRP-3

Pancreatic sections were cut at 5 μm thickness. After coding, hematoxylin and eosin (H & E) and immune-histochemical stains were used for the staining of sections. Xylene was used to dewax sections, then sections were rehydrated, and to inactivate endogenous peroxidase for immunohistochemical staining, sections were incubated in 3% hydrogen peroxide. Sections were incubated for 30 min with 10 mM citrate buffer (PH 6.0) at 95◦C. Sections were incubated with primary rabbit polyclonal AQP-7 antibody (bs-2506R, Bioss, Woburn, USA, at 1:200 dilution) and primary rabbit polyclonal NLRP3 antibody (bs-10021R, Bioss, Woburn, USA, at 1:200 dilution) at 4◦C overnight. The next day, phosphate-buffered saline was used to wash the slides, which were then treated with a secondary antibody. Diaminobenzidine (DAB) (Mouse and rabbit HRP/DAB (ABC) detection IHC kit, ab64264, Abcam, UK) was utilized to stain the slides for determination of immune reactivity, which was detected as a brown color. Sections were counterstained with hematoxylin (Suvarna et al. 2018).

Image analysis and measurements were performed as follows: The positive expression of NLRP3 (Xue et al. 2015; Kong et al. 2017) was considered as the optical density occupied by brown pixels in the examined fields (Tran et al. 2014). For AQP-7, it is measured in the area (390 × 360 micron), representing islets of Langerhans in the examined fields. Five non-overlapping random fields (X400) of three non-consecutive sections for each animal in the groups were chosen and photographed using the Olympus^®^ SC100 digital camera installed on the Olympus^®^ CX41light microscope. Using the instructions of the NIH Image J program (National Institutes of Health, Bethesda, MD, USA), the morphometric study was performed (Mohammed et al. 2023).

For isolation of the color content of each image, color deconvolution plugins were used, and then the brown color was calculated using Image J (Ross 2014). OD was calculated, and the mean gray value was measured as follows: max intensity = 255 for 8-bit images, where OD = log (max intensity/mean intensity) (Del Barrio et al. 2021).

### Statistical analysis

IBM SPSS Statistics for Windows, Version 26.0. (Armonk, NY: IBM Corp) was utilized to analyze the data. To evaluate the quantitative data normality, Shapiro-Wilk’s test was used. Statistical differences involving multiple group comparisons were determined by one-way ANOVA, and differences between different groups were determined with Post-hoc Tukey’s test. The Kruskal-Wallis test is an equivalent test to ANOVA and was used when ANOVA assumptions were violated to compare between more than two groups of skewed data (non-normally distributed data).

## Results

### Effect of alternate day fasting (ADF) and vitamin D on body weight

At the end of the work, group II (HFFD) showed a significant increase in body weight when compared to group I (control) (*P* < 0.001). Group III (HFFD + ADF) and group IV (HFFD + vitamin D) showed a significant increase in body weight when compared to group I (control) (*P* < 0.001) and a non-significant difference when compared with group II (HFFD) (Table 2**).**

**Table 2:** Effect of alternate day fasting (ADF) and vitamin D on body weight

Parameters	G I (Control) (*n* = 6)	GII (HFFD) (*n* = 6)	GIII (HFFD + ADF) (*n* = 6)	GIV (HFFD + vitamin D) (*n* = 6)	Significance test
Body weight (g)	**298 ± 10.3**	**350 ± 74.9**	**380 ± 53.1**	**333.3 ± 50.3**	***P*** < 0.001*

All results are presented as mean ± SD, F: One-way ANOVA *: Statistically significant (*P* ≤ 0.05). *Abbreviations: HFFD: high-fat fructose diet*,* ADF: alternate day fasting*

### Effect of alternate day fasting (ADF) and vitamin D on glucose profile

In this study, there was a significant elevation in fasting blood glucose, serum insulin, and HOMA-IR in group II (HFFD) when compared to group I (control) (*P* < 0.001). Regarding these parameters in other groups, there was a significant decline in group III (HFFD + ADF) and group IV (HFFD + vitamin D) when compared to group II (HFFD) (*P* < 0.001). However, non-significant changes in insulin and HOMA-IR were obtained by intermittent fasting in group III and vitamin D in group IV (Table 3**).**

**Table 3:** Effect of alternate day fasting (ADF) and vitamin D on glucose profile

Variables	G I (Control group) (*n* = 6)	GII (HFFD group) (*n* = 6)	GIII (HFFD + ADF group) (*n* = 6)	GIV (HFFD + vitamin D group) (*n* = 6)	Significance test
Blood Glucose (mg/dl)	109.17 ± 10.01	301.50 ± 28.43	140 ± 7.90	167.33 ± 7.23	F = 168.416 *P* < 0.001*
P1		< 0.001*	0.016*	< 0.001*	
**P2**			< 0.001*	< 0.001*	
**P3**				0.036*	
**Serum Insulin (Pg/ml)**	**272 ± 9.14**	**299.67 ± 12.50**	**212.33 ± 15.69**	**195.17 ± 18.21**	**F = 71.087** ***P*** < 0.001*
**P1**		0.015*	< 0.001*	< 0.001*	
**P2**			< 0.001*	< 0.001*	
**P3**				**0.194**	
**HOMA IR**	**2.10 ± 0.15**	**6.39 ± 0.40**	**2.10 ± 0.11**	**2.31 ± 0.17**	**F = 477.793** ***P*** < 0.001*
**P1**		< 0.001*	**0.999**	**0.442**	
**P2**			< 0.001*	< 0.001*	
**P3**				**0.442**	

All results are presented as mean ± SD, F: one-way ANOVA followed by post hoc Tukey’s test were used to compare between the groups, P1: significance in relation to GI (control group), P2: significance from GII (HFFD group), P3: significance in relation to GIII (HFFD + ADF group), *: statistically significant (*P* ≤ 0.05). *Abbreviations: HFFD: high-fat fructose diet; ADF: alternate day fasting; FBG: fasting blood glucose; HOMA-IR: homeostasis model assessment index*

### Effect of alternate day fasting (ADF) and vitamin D on lipid profile

**Fig. 1:**
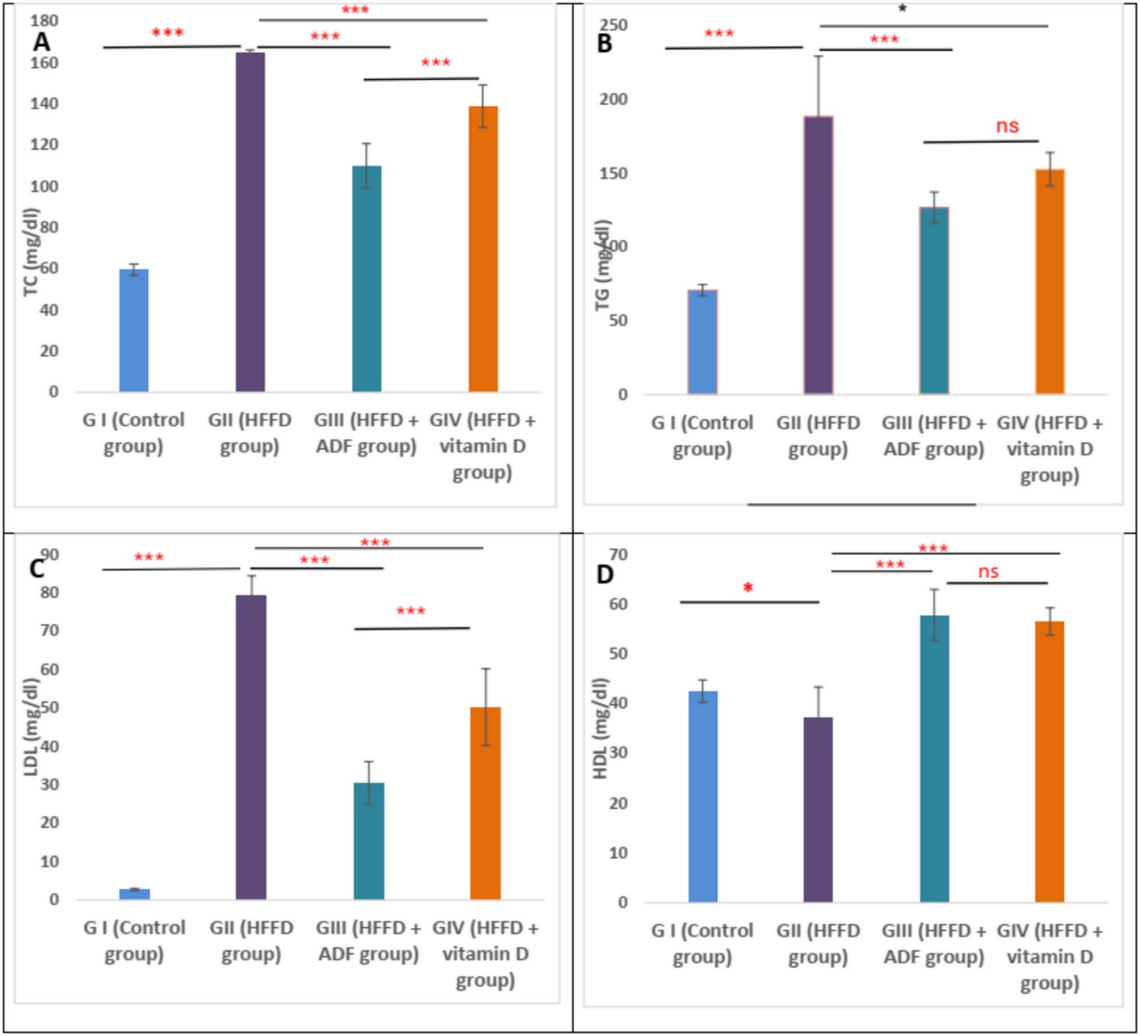
Effect of alternate day fasting (ADF) and vitamin D on lipid profile. All results are presented as mean ± SD. One-way ANOVA followed by post hoc Tukey’s test was used to compare the groups. *Abbreviations: HFFD: high-fat fructose diet; TC: total cholesterol; ADF: alternate day fasting; LDL: low-density lipoproteins; TG: triglycerides; HDL: high-density lipoproteins.* Asterisks indicate significant differences (ns: no significance, *p < 0.05, **p < 0.01, ***p < 0.001)

There was a significant increase in TC, TGs, and LDL-C and a significant decrease in HDL-C (*P* < 0.001) in group II (HFFD) when compared to group I (control). On the contrary, when rats with HFFD were subjected to alternate day fasting (group III) or vitamin D (group IV), there were significant improvements in the lipid profile when compared to group II (rats with HFFD) (*P* < 0.001). In addition, ADF group appeared to have a more significant improvement in the lipid profile than vitamin D treated rats, regarding TC and LDL-C levels (*P* < 0.001) (Fig. 1).

### Effect of alternate day fasting (ADF) and vitamin D on the inflammatory markers

**Fig. 2:**
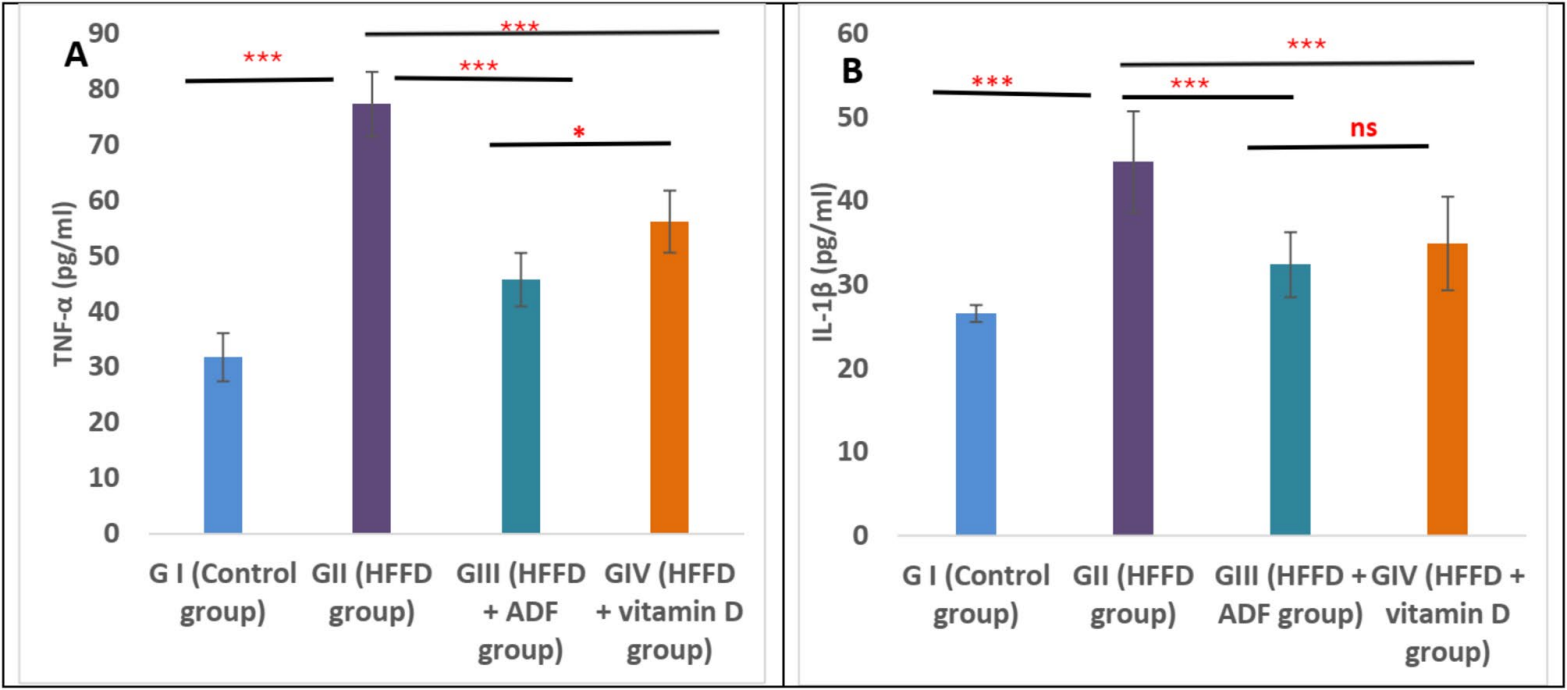
Effect of alternate day fasting (ADF) and vitamin D on inflammatory markers,** including (A) TNF-α**,** and (B) IL-1β measured by ELISA in pancreatic tissues in different studied groups.** All results are presented as mean ± SD. One-way ANOVA followed by post hoc Tukey’s test was used to compare between the groups. *Abbreviations: HFFD: high-fat fructose diet; ADF: alternate day fasting; TNF-α: Tumor necrosis factor-α; IL-1β: interleukin-1β.* Asterisks indicate significant differences (ns: no significance, *p < 0.05, **p < 0.01, ***p < 0.001)

Regarding the inflammatory markers measured in pancreatic tissues, there was a statistically significant elevation in the level of both TNF-α and IL-1β in group II (HFFD) when compared to group I (control) (*P* < 0.001). In group III subjected to alternate day fasting and group IV treated with vitamin D, there was a significant decrease in TNF-α and IL-1β when compared with group II (*P* < 0.001). Moreover, the ADF resulted in a more significant decrease in TNF-α when compared to vitamin D-treated rats (*P* < 0.001) without a significant difference in IL-1β between the two groups (Fig. 2**).**

### Effect of alternate day fasting (ADF) and vitamin D on oxidative stress biomarkers

**Fig. 3:**
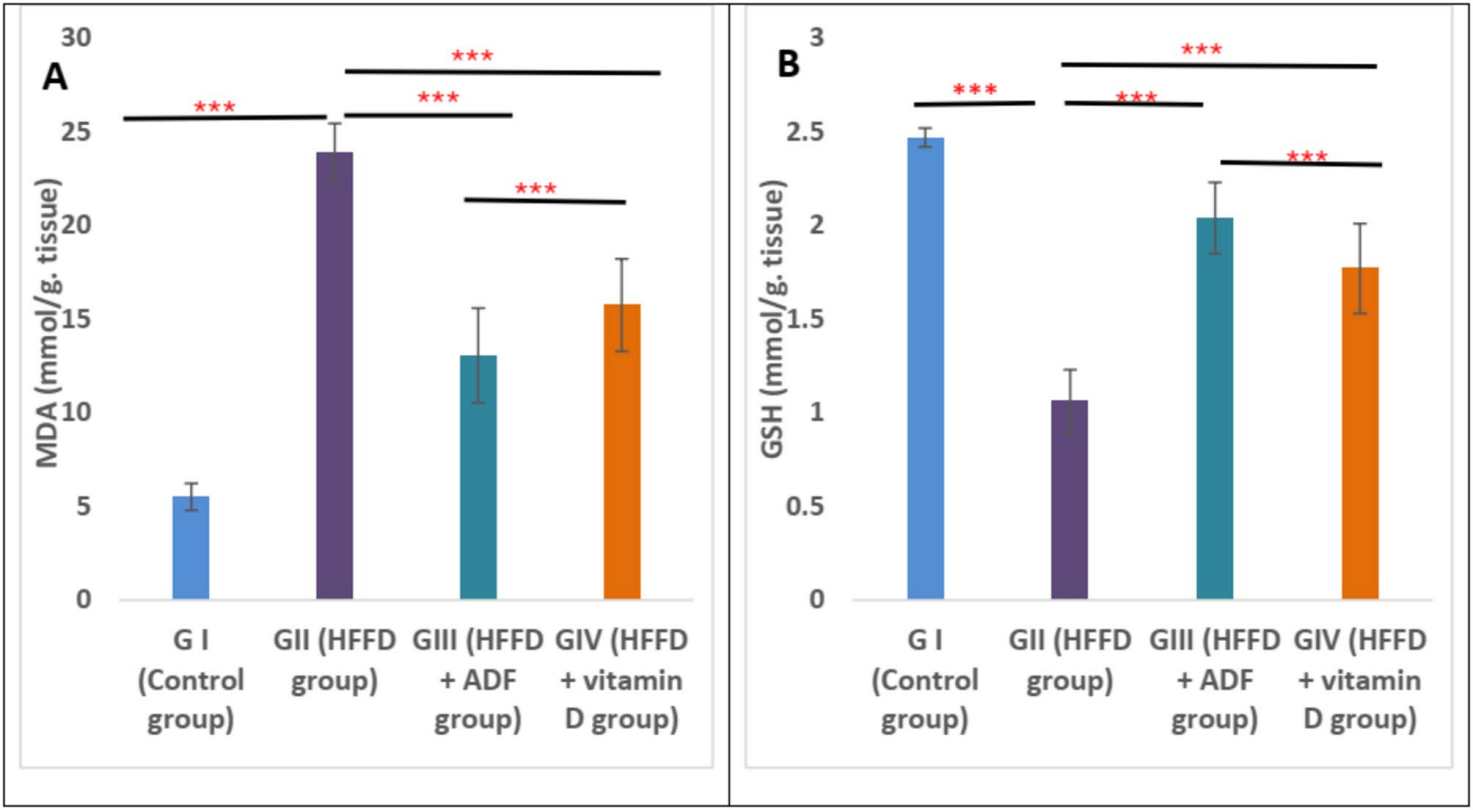
Effect of alternate day fasting (ADF) and vitamin D on oxidative stress biomarkers, including (**A**) malondialdehyde, and (**B**) reduced glutathione measured by colorimeter in pancreatic tissues in different studied groups. All results are presented as mean ± SD. One-way ANOVA followed by post hoc Tukey’s test was used to compare between the groups. *Abbreviations: HFFD: high-fat fructose diet; ADF: alternate day fasting; MDA: malondialdehyde; GSH: reduced glutathione.* Asterisks indicate significant differences (ns: no significance, *p < 0.05, **p < 0.01, ***p < 0.001)

Regarding the oxidative stress biomarkers measured in pancreatic tissues; there was a significant increase in MDA and a significant decrease in GSH in group II (HFFD) when compared with group I (control) (*P* < 0.001). In group III (HFFD + ADF) and group IV (HFFD + vitamin D), there was a significant decrease in MDA along with a significant increase in GSH when compared to group II (HFFD) (*P* < 0.001). Moreover, the improvement in oxidative stress was more prominent in group III (HFFD + ADF) when compared to group IV (HFFD + vitamin D) (Fig. 3**).**

### Effects of alternate day fasting (ADF) and vitamin D on the mRNA expression levels of AQP-1, AQP-3, and AQP-7 in pancreatic tissues

**Fig. 4:**
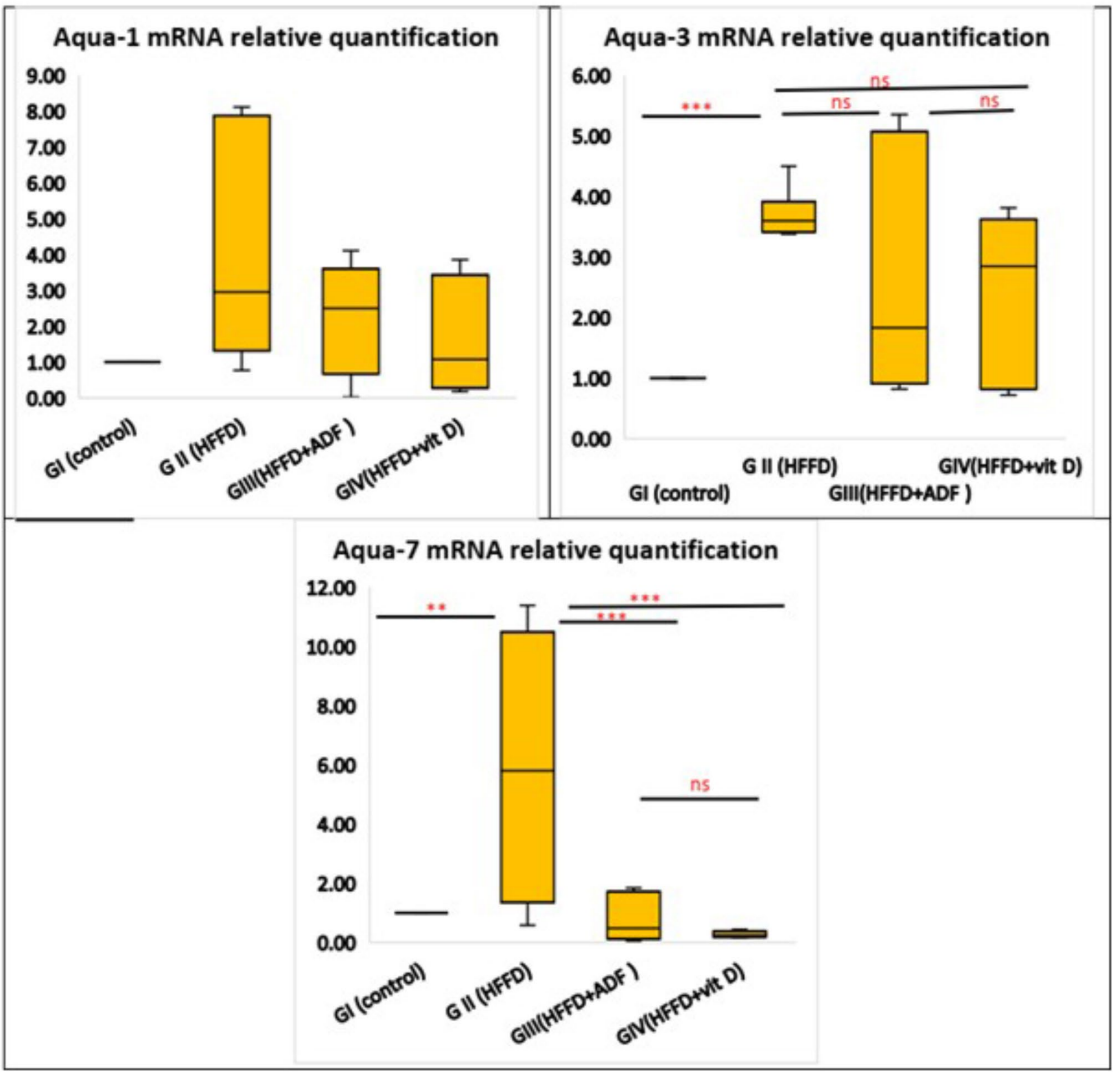
Evaluation of AQP-1, AQP-3, and AQP-7 mRNA expressions among the studied groups. All results are represented as median and interquartile range (IQR), Kruskal-Wallis test followed by the Bonferroni post hoc test was used to compare between the groups. *Abbreviations: HFFD: high-fat fructose diet; ADF: alternate day fasting; AQP: aquaporins.* Asterisks indicate significant differences (ns: no significance, *p < 0.05, **p < 0.01, ***p < 0.001)

The results gained from our study showed that there was no significant change in mRNA expression of AQP-1 between the studied groups (*P* = 0.216); however, AQP-3 (*P* = 0.07) and AQP-7 (*P* = 0.003) were statistically significantly different between the four groups. There was a significant increase in the mRNA expression of AQP-3 and AQP-7 in group II (HFFD) when compared with group I (control). Group III (HFFD + ADF) and group IV (HFFD + vitamin D) showed a non-significant improvement in AQP-3 and a significant decrease in AQP-7 when compared to group II (Fig. 4).

### Histopathological examination of pancreatic tissue specimens

**Fig. 5:**
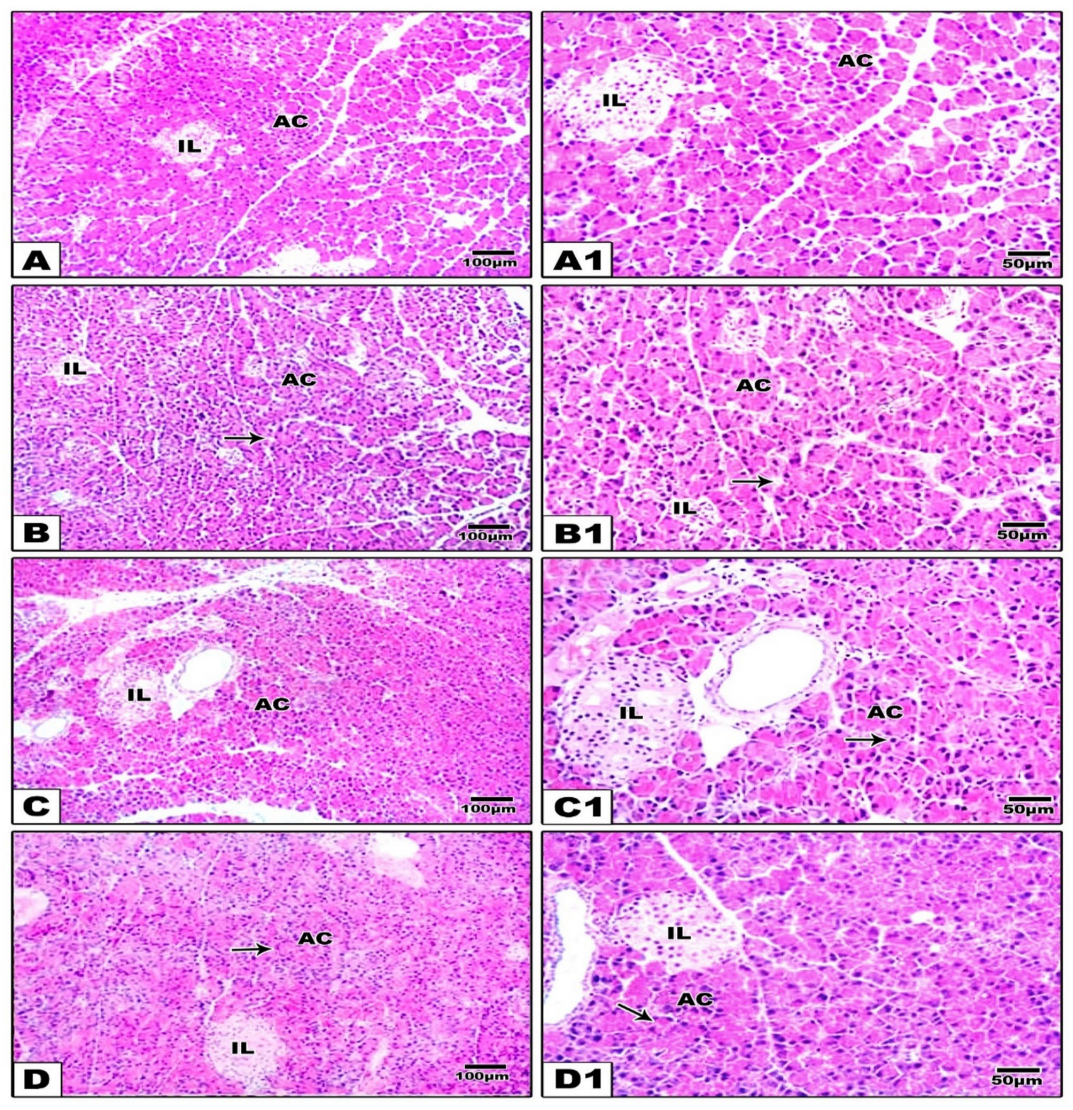
Photomicrographs of pancreatic specimens in the different studied groups. Pancreatic specimens from group I (control) **(A**,** A1)** showed normal pancreatic architecture, including regularly stained acini (AC), with basal nuclei and normal spherical islet cells of Langerhans (IL). Pancreatic specimens from group II (HFFD) **(B**,** B1)** showed pale vacuolated stained cytoplasm in islet cells (IL) and acinar cells (AC) with irregular arranged nuclei (black arrows). Group III (HFFD + ADF) **(C**,** C1)** revealed eosinophilic stained cytoplasm with basal nuclei in acinar cells (black arrow) nearly near the control and nearly normal islet cells (IL). Group IV (HFFD + vitamin D) **(D**,** D1)** showed eosinophilic stained cytoplasm with no fat vacuoles in acinar cells (AC)and nearly normal basal nuclei (black arrow) and nearly normal islet cells (IL) (low magnification lens X; 100 bar 100 with high magnification lens X; 200 bar 50)

The H & E-stained pancreatic sections of group I (control) revealed normal pancreatic architecture, including regularly stained acini, duct, and islet cells of Langerhans (Fig. 5A, B**).** On the other hand, pancreatic tissues of rats with HFFD showed pale vacuolated stained cytoplasm in acinar cells (Fig. 5C, D**).** Furthermore, specimens from the HFFD subjected to either ADF (Fig. 5E, F) or vitamin D (Fig. 5G, H) showed eosinophilic stained cytoplasm in acinar cells nearly near the control with no fat vacuoles in acinar cells.

### Effects of alternate day fasting (ADF) and vitamin D on the Immunohistochemical analysis of AQP-7 in pancreatic tissues

**Fig. 6:**
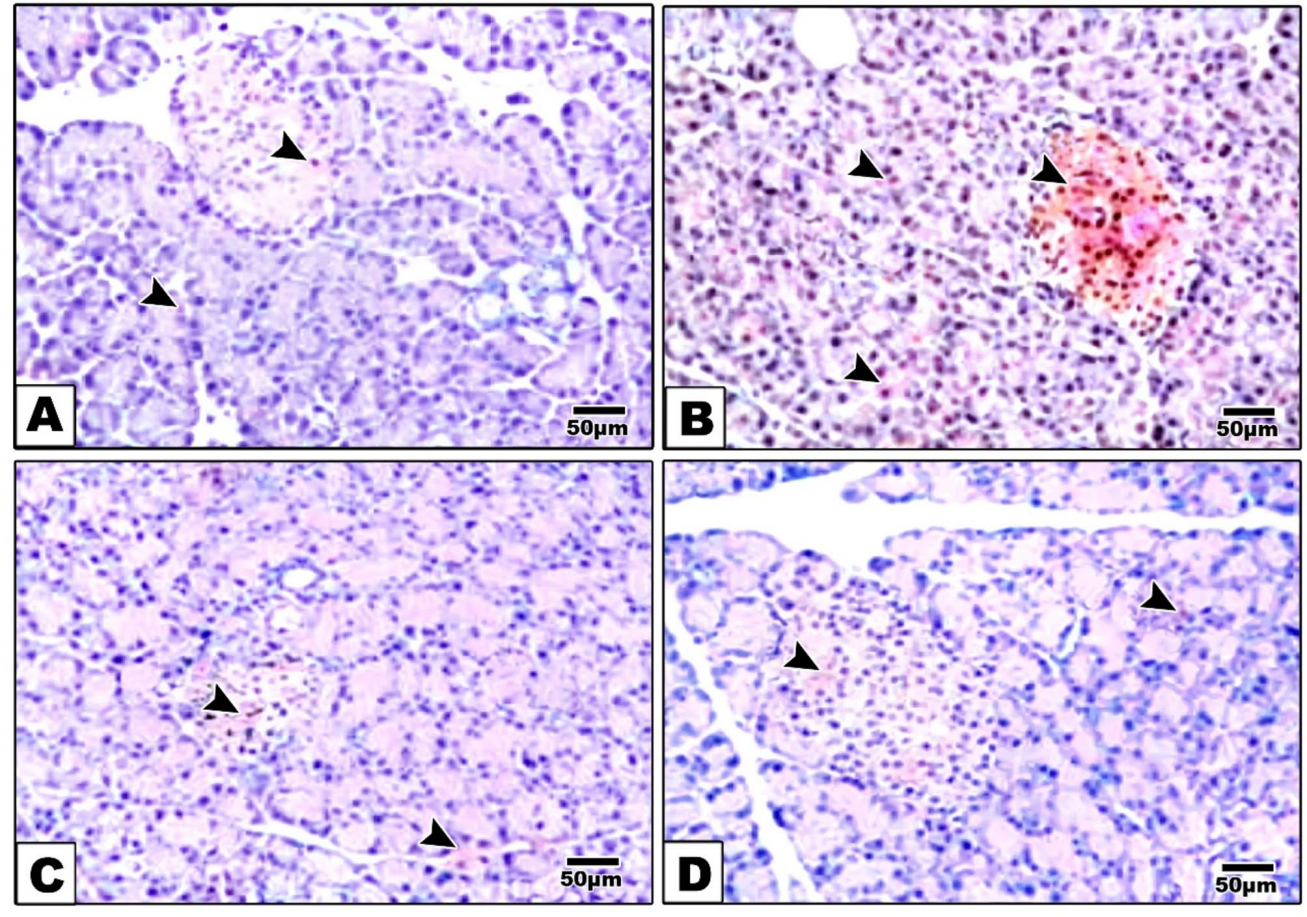
Microscopic examination of immune-stained pancreatic tissues against AQP-7. Group I (basal diet) (**A**) showed weakly stained Langerhans islets and acini (arrowhead). Group II (HFFD) group (**B**) showed intense brown staining acini and in Langerhans islets (arrowhead). Group III (HFFD + ADF) (**C**) revealed mild staining in acini and Langerhans islets (arrowhead) Group IV (HFFD + Vitamin. D) (**D**) showed very faint cytoplasmic staining in acini and in Langerhans islets (arrowhead). (High magnification lens X; 200 bar 50)

Immunohistopathological examination of pancreatic sections stained with AQP-7 in group I (control) showed weakly stained islets of Langerhans (Fig. 6A, B). In contrast, pancreatic tissue from group II (HFFD) (Fig. 6C, D) showed intensely stained brown Langerhans islets. Furthermore, specimens from the HFFD treated with intermittent fasting (Fig. 6E, F) revealed minimal brown staining in Langerhans islets. Moreover, the specimens from HFFD treated with vitamin D (Fig. 6G, H) showed faint brown staining Langerhans islets.

### Effects of alternate day fasting (ADF) and vitamin D on the immunohistochemical analysis of NLRP3 in pancreatic tissues

**Fig. 7:**
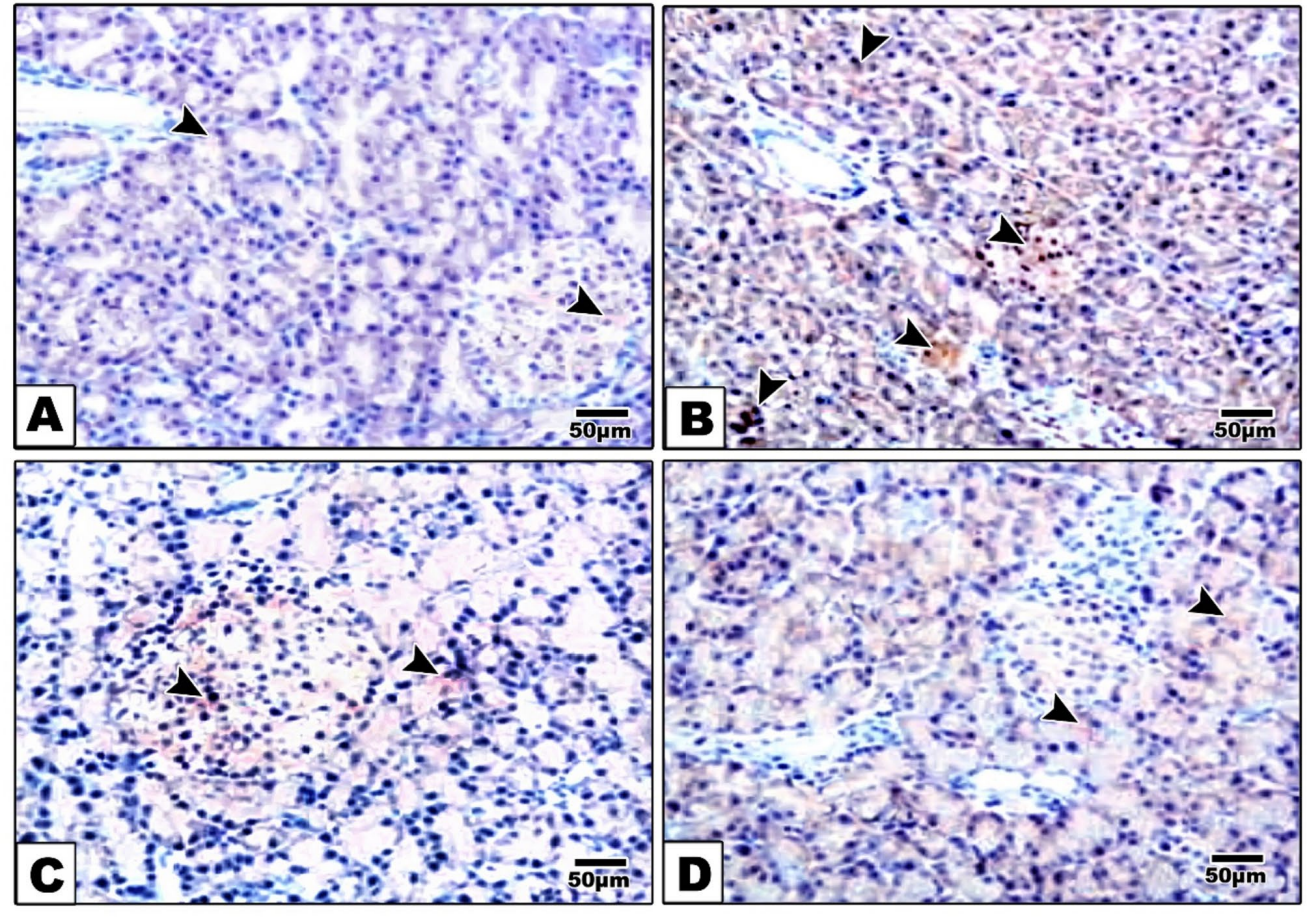
Microscopic examination of immune-stained pancreatic tissues against NLRP3. Group I (control) (**A**) showed poorly stained acini and Langerhans islets (arrowhead). Group II (HFFD) (**B**) showed intense brown cytoplasmic granulated staining in acinar cells and Langerhans islets (arrowhead). Group III (HFFD + ADF) (**C**) revealed mild cytoplasmic staining in acinar cells and Langerhans islets (arrowhead). Group IV (HFFD + vitamin D) (**D**) showed faint cytoplasmic staining in acinar cells (arrowhead). (High magnification lens X; 200 bar 50)

To assess the mechanism behind the alteration in pancreatic endocrine function with high fat fructose diet, the immunohistochemical examination of NLRP3 as a marker of inflammasome activation was done in the different studied groups (Fig. 7). Group I (control) showed pancreatic tissues with poor stained acini and Langerhans islets (Fig. 7A, B**).** Group II (HFFD) showed intense brown cytoplasmic granulated staining in acinar cells and Langerhans islets (Fig. 7C, D**).** Group III (HFFD + ADF) revealed mild cytoplasmic staining in acinar cells and Langerhans islets (Fig. 7E, F**).** Group IV (HFFD + vitamin D) showed faint cytoplasmic staining in acinar cells and Langerhans islets (Fig. 5G, H**).**

### Effects of alternate day fasting (ADF) and vitamin D on the relative protein expression of AQP-7 and NLRP3 in pancreatic tissues

**Fig. 8:**
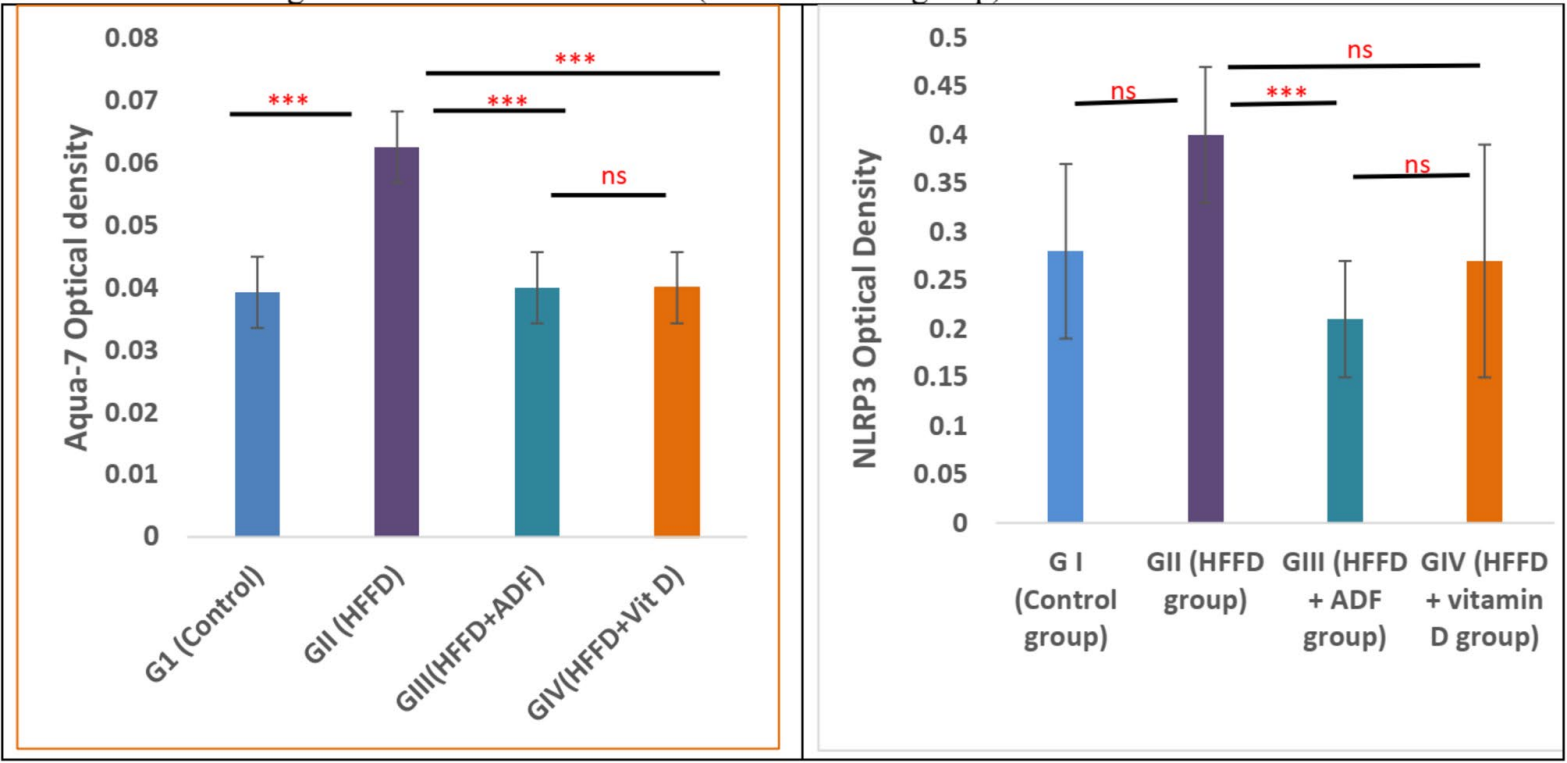
Effect of alternate day fasting (ADF) and vitamin D on the relative protein expressions of (**A**) AQP-7, and (**B**) NLRP3 in pancreatic tissues in different studied groups. All results are presented as mean ± SD. One-way ANOVA followed by post hoc Tukey’s test was used to compare between the groups. *Abbreviations: HFFD: high-fat fructose diet; ADF: alternate day fasting; AQP: aquaporins; NLRP3: Nucleotide-binding domain*,* leucine-rich–containing family*,* pyrin domain–containing-3.* Asterisks indicate significant differences (ns: no significance, *p < 0.05, **p < 0.01, ***p < 0.001)

In our experiment, the relative protein expression of AQP-7 was measured by immune staining, inwhich the histogram illustrated the mean optical density of AQP7, which revealed that a significant elevation in AQP-7 expression was observed in group II (HFFD) when compared to group I (control) (0.062 ± 0.017, 0.039 ± 0.026, respectively; *P* = 0.002). On the other hand, its expression was significantly decreased in group III (HFFD + ADF) (0.04 ± 0.015) and group IV (HFFD + vitamin D) (0.04 ± 0.016) when compared to group II (HFFD) (*P* < 0.001) (Fig. 8A**).** There was a significant elevation in the relative protein expression of NLRP3 in group II (HFFD) when compared to group I (control) (*P* < 0. 01). However, its expression was significantly attenuated in group III (HFFD + ADF) and group IV (HFFD + Vitamin D) when compared to group II (HFFD) (*P* < 0.001) (Fig. 8B).

## Discussion

The mechanisms behind pancreatic steatosis remain poorly understood. It may be due to either adipocyte replacement of acinar cells due to underlying pancreatic diseases or pancreatic ectopic fat deposition due to obesogenic diets (Wagner et al. 2022).

Aquaglyceroporin was shown to have a direct role in metabolic-related disorders such as pancreatitis and type II diabetes (da Silva et al. 2020). Our study was designed to assess the potential role of aquaporins in the pathogenesis of pancreatic steatosis in the rat model of HFFD and to explain the mechanisms behind the therapeutic benefit of ADF and the supplementation of vitamin D in ameliorating pancreatic steatosis.

Our study showed that the histopathological changes observed in rats receiving HFFD confirmed the development of pancreatic steatosis in the form of fatty infiltration of acinar cells with pale vacuolated stained cytoplasm. These results were in line with a previous study performed by Zhao et al. (2022), which demonstrated a decrease in islet size, irregular morphology, and infiltration of pancreatic tissues by many adipocytes. On the other hand, the ADF and vitamin D treatment resulted in improvement of the condition as detected by eosinophilic stained cytoplasm in acinar cells with no fat vacuoles in acinar cells.

In the current study, administration of HFFD for 12 weeks resulted in an increase in body weight compared to the control group, in line with results of studies performed by Wu et al. (2017) and Lowry et al. (2019). Weight gain can be attributed to extra calorie intake, which promotes positive energy balance (Lazar et al. 2019).

The body weight of rats fed HFFD + ADF was insignificantly different in comparison to the HFFD group. This finding is in line with Abbasi et al. (2020). This could be justified as the ADF group of rats may have adopted a gorging eating pattern, which can change normal nighttime feeding to a pattern of overfeeding during daylight hours (Harvie and Howell 2017).

Contrary to our finding, Wilson et al. (2018) showed that regardless of exercise, fasting for 12 weeks resulted in less weight gain. Also, the body weight of rats fed HFFD simultaneously with vitamin D was insignificantly different in comparison to the HFFD group, and this is in line with the studies of Karampela et al. (2021) and Marziou et al. (2020), which found that 15 weeks of vitamin D supplementation had no impact on obesity. However, Benetti et al. (2018) reported that obese rats treated with 1,25(OH) 2D showed a limited increase in weight compared to the control obese group.

The lack of effect of vitamin D on body weight could be due to a combination of factors, including metabolic and hormonal resistance, inadequate dosing or duration of vitamin D, and the complexity of obesity-related dysregulation of vitamin D metabolism (Bonnet et al. 2019). Vitamin D might still influence other metabolic processes (such as insulin sensitivity or inflammation), but these changes may not directly result in weight loss.

Regarding the glucose profile, our study demonstrated that 12 weeks of HFFD resulted in a significant elevation in FBG, serum insulin, and IR. This came in line with results of studies performed by Moughaizel et al. (2022) and Guney et al. (2023), as the high-fat diet and the lipogenic effect of fructose stimulate the deposition of visceral fats, leading to the accumulation of lipid in insulin-sensitive tissues, impairing insulin signalling, and increasing the flux of fatty acids.

The current study showed that the HFFD + ADF group demonstrated a significant decrease in FBG, serum insulin, and IR. These results are in line with the results of a previous study, and this might be attributed to the inhibition of inflammation and the NLRP3-inflammasome (Liang et al. 2021). Furthermore, vitamin D supplementation improved the previous parameters, and these findings came in agreement with the study of Talaei et al. (2013). Vitamin D preserves the mass of the B cells of the pancreas and stimulates the expression of insulin receptors by inhibiting inflammation (Angellotti and Pittas 2017).

Regarding the lipid profile, the present work showed that the HFFD for 12 weeks resulted in dyslipidemia, as shown by increased serum levels of TG, TC, and LDL along with a decline in HDL levels. This resultcould be due to excess exogenous lipids derived from a HFD in addition to excess de novo lipogenesis triggered by a high-fructose diet (Moughaizel et al. 2022). These results are in line with the study of Benyelles et al. (2023), which demonstrated that a high-fructose diet resulted in dyslipidemia, hyperglycemia, hyperinsulinemia, and oxidative stress (Benyelles et al. 2023).

The application of ADF or supplementation of vitamin D to the HFFD rat group had valuable effects on the lipid profile. These results come in line with the previous study performed by Surdu et al. (2021). Furthermore, the study performed by Marinho et al. (2019) confirmed that ADF improved the lipid profile and diminished plasma levels of TG and TC.

Regarding oxidative stress, our study illustrated that HFFD for 12 weeks resulted in prominent oxidative stress in pancreatic tissues, as demonstrated by increased MDA levels along with a significant decrease in GSH. These results are in line with the results of Zhao et al. (2022) and Guney and Akar (2023). The ADF and vitamin D supplementation resulted in a significant decline in MDA and an elevation in GSH levels. This result was previously reported by a study performed by Marinho et al. (2019), which demonstrated that the ADF had antioxidant and anti-inflammatory activity in mice fed HFFD. A previous study done by Sepidarkish et al. (2019) demonstrated that vitamin D can ameliorate oxidative stress through the upregulation of GSH in the cells and antioxidant enzymes such as superoxide dismutase and glutathione peroxidase.

In the current study, the levels of inflammatory cytokines, including IL-1β and TNF-α, were investigated with a significant elevation in the levels of IL-1β and TNF-α in rats receiving HFFD. These results agree with the results of Zhao et al. (2022). The consumption of HFFD induces ROS that triggers proinflammatory signaling and stimulates nuclear factor kappaB (NF-κB), with subsequent upregulation of NF-κB-dependent proinflammatory molecules such as interferon-γ (IFN-γ) and TNF-α (Tan and Norhaizan 2019).

HFFD-treated rats with either ADF or vitamin D showed a significant decrease in these inflammatory parameters, whereas ADF appeared to have more valuable effects than vitamin D. Previous studies have also reported that ADF decreases the number of inflammatory markers such as TNF-α (Lavallee et al. 2022; Guney and Akar 2023). Moreover, previous studies confirmed that vitamin D has anti-inflammatory activity in many pathological conditions of the pancreas. The suggested underlying mechanism is that vitamin D negatively modulates the release of IL-6 and TNF-α (Cai et al. 2022).

The AQP1 has a cytoprotective role and anti-inflammatory effect as it decreases the expression of TNF-α, IL-1β, and NF-κB (Kandemir et al. 2018). However, our study failed in finding a significant difference in the mRNA expression level of AQP1 between the control, the HFFD group, and the HFFD treated with either ADF or vitamin D.

In our study, the AQP-3 and − 7 expression levels were considerably higher in the HFFD rat group than in the control group, whereas treatment with either ADF or vitamin D resulted in decreased expression of AQP-3 and − 7 in pancreatic tissues.

In addition, the increased insulin level in parallel with the increased AQP-7 expression level in rats receiving HFFD for 12 weeks might point to the trial of the β-cell of the pancreas to compensate for the hyperglycemia and increased insulin resistance in the HFFD rat group by producing elevated levels of insulin, which can be caused by increased intracytoplasmic content of glycerol followed by rapid β-cell re-swelling, activation of anion channels that are volume-regulated, and subsequent secretion of insulin (Best et al. 2009).

This finding is in line with another study where a significant elevation in AQP-3 and − 7 expression levels was found in the rat jejunum in the HFFD group and a significant decline in AQP-3 and − 7 expression levels was detected with intermittent fasting (Elhessy et al. 2024). These results are contradicted by the results of Méndez-Giménez et al. (2018), which reported that AQP-7 upregulation in β-cells after bariatric surgery participates in improving insulin secretion and decreasing pancreatic steatosis by elevating the glycerol content intracytoplasmic in obese rats.

Moreover, the AQP-7 knockout mice showed hyperinsulinemia, decreased size and mass of beta cells, increased glucose-induced and basal insulin release, and increased triglycerides and glycerol contents (Louchami et al. 2012). However, another study found that AQP-7 knockout mice expressed decreased glucose-stimulated and basal insulin secretions (Arsenijevic et al. 2019). Arsenijevic and his colleagues explained these discrepancies using different methodologies and/or different mice’s genetic backgrounds.

The present study demonstrated increased expression of NLRP3-inflammasome protein in pancreatic tissues of rats receiving HFFD for 12 weeks. The metabolic changes resulted from HFFD were reported as a danger signal to stimulate the NLRP3-inflammasome complex, which explains the high levels of IL-1β and TNF-α (Kelley et al. 2019; Neudorf and Little 2023). There is a mutual interplay between IL-1β and NLRP3, in which the increased level of IL-1β is needed for the activation of the NLRP3-inflammosome. Active NLRP3 leads to the activation of caspase-1, which subsequently cleaves pro-IL-1β into its active forms. IL-1β is a pro-inflammatory cytokine that can cause the release of other inflammatory cytokines, such as TNF-α (Arend et al. 2008).

In contrast, both the ADF and vitamin D groups demonstrated weak positive staining against NLRP3 and decreased pancreatic tissue levels of IL-1β and TNF-α. These results agree with Liang et al. (2021) and Cao et al. (2020), who demonstrated that vitamin D is a key regulator of immunity and plays a major role in regulating NLRP3-inflammasome activity. Vitamin D, by acting on its receptors, can inhibit the activation of NLRP3.

The link between inflammation and aquaporin expression in pancreatic tissues still needs more clarification. Recently, cell re-swelling and AQP-mediated water influx have been considered as a step that precedes NLRP3 stimulation and IL-1β release (Kalita and Das 2024*).* Blockage of AQP3 reduced IL-1β release by preventing NLRP3 inflammasome activation induced by re-swelling and ATP (da Silva et al. 2021). Therefore, AQPs potentially represent a target for new therapeutic approaches to alleviate the histopathological, biochemical, and molecular changes of pancreatic steatosis related to HFFD.

One of the limitations of our study is that we did not assess body mass index (BMI) and visceral fat, which may have provided additional insights into the relationship between HFFD, ADF, and vitamin D supplementation and overall metabolic health. Future research should consider including BMI and visceral fat measurements to better understand potential associations. Moreover, our study did not assess fatty infiltration of the pancreas using specialized staining techniques such as Oil Red O, which could have provided additional insights into the extent of lipid accumulation within pancreatic tissues. Furthermore, we did not evaluate the insulin immunohistochemical staining, which would have allowed for a more detailed assessment of pancreatic endocrine function.

The promising results of ADF and vitamin D in improving pancreatic function and reducing inflammation open the door for future clinical trials aimed at evaluating their combined effects in patients with metabolic syndrome or prediabetes. The additive or synergistic effects of such therapies could provide a novel approach to managing metabolic and inflammatory diseases.

## Conclusions

In conclusion, HFFD resulted in metabolic alterations both in glucose and lipid metabolism, together with increased pancreatic tissue inflammation, oxidative stress, and increased expression of the NLRP3-inflammosome, AQP-3, and AQP-7. Treatment with either ADF or vitamin D appeared to mitigate the effects of HFFD on pancreatic tissue structure and function with improvements in metabolic parameters, decreased inflammation, and oxidative stress, with a more favourable effect of ADF.

## Data Availability

No datasets were generated or analysed during the current study.

## Abbreviations

ADF: Alternate day fasting
AQPs: Aquaporins
CT: Cycle threshold
FBG: Fasting blood glucose
GAPDH: Glyceraldehyde-3-phosphate dehydrogenase
GSH: Reduced glutathione
HDL-C: High density lipoprotein-cholesterol
HFFD: High fat fructose diet
IF: Intermittent fasting
IL-1β: Interleukin-1β
IR: Insulin resistance
LDL-C: Low density lipoprotein -cholesterol
MDA: Malondialdehyde
NLRP3: Nucleotide-binding domain, leucine-rich–containing family, pyrin domain–containing-3
qRT-PCR: Quantitative real-time polymerase chain reaction
TC: Total cholesterol
TGs: Triglycerides
TNF-α: Tumor necrosis factor-α
